# Progressive Locomotor Ataxia, or Duchenne’s Disease

**Published:** 1869-07

**Authors:** Stanhope Breckinridge

**Affiliations:** Professor of Materia Medica and Therapeutics, Louisville, Ky.


### Miscellaneous.

### Progressive Locomotor Ataxia, or Duchenne's Disease.

By STANHOPE BRECKINRIDGE, M. D., Professor of Materia Medica and Therapeutics, Louisville, Ky.

The symptoms of this most interesting malady, as found described in Trousseau's clinical medicine, Victor Baziré's report of eight cases, and the article on this affection in Reynolds' system of medicine, are briefly as follows, viz.: *pain*, partial paralysis of the bladder, paralysis of some of the muscles regulating the movements of the eye-ball, a sense of constriction in different parts of the body, loss of cutaneous sensibility, and sometimes anæsthesia of the mucous membranes, and of the muscles and articular surfaces; and lastly, and most important of all, the loss of the power of coördinating muscular movements.

This latter symptom is so prominent that it has given the disease its name. When an ataxic patient attempts to walk, he staggers worse than a drunken man, and rolls from side to side in endeavoring to maintain his equilibrium; his legs fly out from under him, and jerk about with indescribable confusion, and unless he supports himself upon a cane, or other prop, he often falls to the ground. Before the affection reaches this advanced stage, the patient may walk alone, but in a jerky and very disordered manner, particularly when he first starts, and when he attempts to turn round. And yet this irregularity of gait is not dependent on paralysis of the cord, or weakness of the muscles, as was formerly supposed, for Dr. Duchenne (de Bologne) has demonstrated that the muscles retain all, or nearly all, their normal strength, and that the supposed paralytic is usually able to take his medical attendant upon his back and carry him across the ward, if he is allowed to guide himself by the arm of a friend. When these patients are sitting or lying down, it is often impossible to extend or to flex their limbs against their will, showing that their muscular strength is not impaired, but that they have simply lost the power of *coördinating* action.

This progressive irregularity of movement is preceded usually, though not always, by all or part of the symptoms enumerated above. One of the earliest of these is *pain*, and pain of a peculiar and almost pathognomonic character. It comes in lightning-like flashes, and vanishes as quickly as it had come; or it may be of a *boring* nature, and confined to a circumscribed spot. Later in the course of the disease, these pains become continuous, and cause the most acute and unremitting anguish. Heretofore they have been mistaken for neuralgia—technically speaking—and neuralgic rheumatism, but Dr. Duchenne has shown that they are symptomatic of approaching ataxia.

Another early symptom is partial paralysis of the bladder. The fundus of the organ is first affected, but the sphincter may ultimately become involved.

In nearly half of Trousseau's fifty odd cases, there was spermatorrhœa. It, however, occurred in only one of Baziré's eight cases, and it is not supposed to have any intimate connection with the disease. In the early stage of ataxia, some of the patients possess the power of repeating the act of coitus with unexampled frequency, but they all generally become impotent as the malady grows worse. It was observed by Trousseau that men who possess this semblance of exaggerated virile power are often subject to spermatorrhœa.

The gradual or sudden appearance of strabismus is a common symptom as a prelude to the incoördination of muscular power. The strabismus may be either external or internal, and it may or may not be accompanied by diplopia or ptosis. These symptoms may pass away in a few days, or last for the remainder of the patient's life. The sense of constriction which the ataxic individual often feels is at times very distressing. The patient's shoes feel too tight, and his arms, or chest, or legs feel as if they were girdled by a stout band.

One of the most serious of all the symptoms is the cutaneous anæsthesia, which often becomes more or less general, and involves the deep-seated tissues. When the loss of muscular and articular sensibility is added to the cutaneous anæsthesia, the patient cannot feel the resistance of the ground, and he must keep his eyes fixed upon his legs when he attempts to walk, or he will immediately fall. For some time after attention was first directed to this disease, the loss of sensibility was so uniformly observed, and was such a serious phenomenon, that some writers regarded it as the cause of incoördination of movements, but several cases have occurred in which the progressive locomotor ataxia was most distinctly marked, and yet in which there was a complete absence of impaired sensibility. As Trousseau remarks, these cases prove that cutaneous and muscular anæsthesia are only secondary phenomena of the disease. It should be observed that the sensibility to *atmospheric changes* never become impaired.

The malady just described usually attacks persons between the ages of twenty and forty years, though it is occasionally seen far out of these limits. One case is reported at fifteen years and another at eighty, but these are very exceptional.

As far as statistics have yet been collected, females are much less liable to this disease than males, the proportion being about as 1 to 4 or 1 to 6.

*Differential Diagnosis.*—It is scarcely possible for one familiar with all the ordinary symptoms of locomotor ataxia to confound it with any other disease. It has, however, been mistaken for paraplegia, for it generally attacks the lower extremities first, and it may be accompanied by paralysis of the bladder and rectum. The characteristic pain of ataxia and unimpaired muscular power will readily distinguish it from this affection. Cases have been published in which tumors of the cerebellum produced a certain form of ataxia.

Trousseau quotes a case from Herard, (one of the celebrated authors of the latest and best French work on phthisis,) in which the patient had lost the power of associating, combining and coördinating the movements necessary in locomotion, standing, etc. There was no paralysis of sensation or motion. This case resembled quite closely one of ataxia, but it was diagnosed "cere-bellar tumor," on account of the very frequent vomiting which occurred, for this is a symptom, not only foreign to ataxia, but one which, on the authority of Hillairet, is common in cere-bellar diseases. There is very little danger of confounding this malady with other forms of paralysis, or with St. Vitus' dance.

*Pathological Anatomy.*—Post-mortem examinations generally reveal degeneration of the posterior columns of the spinal cord in the dorsal and lumbar regions, and of the roots of these columns. In a few cases the anterior and lateral columns have been found involved in the degenerative process, and Dr. Duchenne reports a case in which "the encephalon and spinal cord, on the most careful examination, presented no anatomical lesion appreciable by the naked eye." The morbid changes are described as consisting in "a kind of gray degeneration, and sometimes in a gelatiniform and translucent condition, in a diminution of consistency, or in a state of induration." In the majority of cases heretofore examined, the posterior columns have been found diminished in size, though in several instances they exceeded the normal volume.

Dr. Axenfeld gives the following resume of the microscopical appearances:

"In the *white matter* of the posterior columns, which has now become yellowish or gray, are seen scattered nerve-tubes, pale, shrunken, or varicose, sometimes reduced to their neurilemend only, or filled with granular contents, a few still retaining their cylinder axis. On the other hand, the connective transparent substance (the *neuralgia* of Virchow,) the blastoderma in which these tubes are imbedded, has become fibrillated, and presents amidst a large quantity of amorphous granules, a smaller quantity of elongated nuclei, and a smaller one still of cells. Corpora amylacea, also, are met with in variable quantity, distinguishable by their usual reaction with tincture of iodine. Lastly, the blood-vessels are considerably developed, and their thickened walls, composed of several layers, are encrusted with a deposit of fatty granules.

In the posterior cornua of the *gray matter* the same alterations are found, but less markedly. The reddish tint of this part is due to the injection of its capillary network and occasionally its tint is darker, even blackish, owing to the presence of numerous granules of pigment. The nerve-tubes in these cornua are sometimes destroyed, and the nerve-cells altered in shape, although in general, both the tubes and cells are normal.

The changes noticed in the posterior roots are the same as those of the corresponding columns; and they are the same again in the diseased portions of the bulb, the pores, and the optic nerves.

On the whole, all these alterations clearly point to *atrophy* of the nervous tissue.

In a case of Trousseau's, the post-mortem showed, among other lesions of the posterior columns, that the nerve-tubes had been mostly destroyed and that nothing was left of them but their sheaths.

And in another case (the examination being conducted by Mr. Sappey,) the posterior cords (in the lumbar region) were very considerably atrophied and the roots had lost two-thirds or three-fourths of their normal size. Their aspect was modified; they were not white, but of a reddish-gray color, looking like bundles of capillary blood-vessels. These lesions are in striking contradiction to what had been anticipated, for it is the accepted physiological belief (considered to be well demonstrated) that the *anterior* columns of the spinal cord control muscular motion.

When we reflect, however, on the true nature of this disease we see that there is no real *paralysis* in it, and consequently that the anterior columns ought not to be affected. The very fact that they remain healthy in many of the worst cases of locomotor ataxia is another argument going to prove that the trouble consists, not in a loss of muscular power, but in a lack of coördination.

This absence of coördinating power, as we remarked above, does not arise from cutaneous or deep insensibility, for several cases have been reported in which the sensibility was entirely normal. We have all been taught to regard the posterior columns as the ones which govern sensation, but it is very difficult for us to understand how sensibility should have remained intact when we see at the autopsy these columns more or less completely degenerated.

Indeed, the experiments of Brown-Séquard, Türck, Philippeau and Vulpian, show that sensibility may remain normal, even after division and removal of a portion of the posterior columns. As Trousseau remarks, this obscurity is only an additional proof that physiology has not said its last word in regard to the functions of the spinal cord. That the posterior columns influence the *coördination of movements* we may feel pretty sure. The pathology of locomotor ataxia is a powerful argument in favor of this view, and the advocacy of it by such eminent phyiologists as those just mentioned almost establishes it as a fact.

As to the nosological classification of this affection, all writers upon it are not agreed. While some consider it to be an idiopathic local disease, Trousseau maintained, till death, his first opinion, uttered years ago, that it belongs really to the class of neuroses.

In other words, the question is whether the material lesions, the degeneration, etc., of the posterior columns, constitute the disease, or whether they are symptoms of it. There can scarcely be a doubt that the spinal lesions produce, in a large measure, the most striking symptom of the disease, the incoördination of movements, but it is urged that they do not account for other and important symptoms, such as pain, and perhaps, cutaneous insensibility. Again, it is true, that the characteristic lesions of the malady are not constant and uniform, for in a few cases where ataxia had been present for years, the post-mortem examination failed to reveal either to the naked eye or to the microscope, the slightest alteration of the posterior columns and roots. The following case is an example, viz.:

A man, aged 44 or 45, had, twelve years before, suffered from the characteristic pains of locomotor ataxia. After three years they left, and were followed by paralysis of the third nerve on the left side, which persisted until death. Amblyopia followed next, ending in double amaurosis, with atrophy of the optic disc. Later, incoördination of movements supervened, and lastly, loss of sexual power. He finally died of small-pox, during the course of which he was attacked by general paralysis, of that kind which occurs in acute diseases, and which has been well described by Dr. Gubler.

Two weeks before his death, Dr. Duchenne examined the patient, and pronounced the case a typical one of locomotor ataxia. There was every reason to expect gelatiniform degeneration of the posterior columns and atrophy of the posterior roots, at the autopsy; but to the surprise of all, the most careful microscopical examination did not reveal these lesions. The *spinal cord* was *generally injected*, but this, it was thought would, at most, only account for the general paralysis which preceded death. The *optic nerves* had a gelatinous aspect, and under the microscope their tubes were seen to be atrophied; the notor oculi on the left side was slightly atrophied also. These were the only anatomical lesions found. Microscopical examination of the cord and its roots, made by Drs. Gubler, Luys and Duchenne, detected no alteration in the posterior columns and roots. On the contrary, when Dr. Duchenne examined transverse sections of the *anterior* lumbar and cervical roots, he found that about one-third of their tubes had disappeared. (Trousseau's clinical lectures, page 179.) Cases like this—even though they be few in number—strengthen the position of those who claim that the post-mortem appearances are not the *cause*, but the *effect* of the disease.

Duchenne and Trousseau incline to the belief that the essence of the trouble in locomotor ataxia lies in the sympathetic nerve, but at present its origin and nature remain under a veil.

Nos. 957 and 959, of the *London Medical Times and Gazette*, of date October 31st and November 14th, 1868, contain papers on "diseases of the joints, connected with locomotor ataxia," by Dr. Benjamin Ball, Prof. Agrégé at the Paris Faculty of Medicine.

Eleven cases are reported of locomotor ataxia complicated with what he calls "arthritis," affecting respectively the knee, shoulder, and elbow joints. The articulating joints swell—sometimes enormously; synovial fluid is poured out; the synovial tissues become more or less disorganized, and the bones rub against each other with a distinct crepitation. The osseous structure becomes absorbed, and the whole appearance of the articulating surface altered. Singularly enough, there is no accompanying *heat* or *pain*. This local manifestation of disease always occurs suddenly.

Dr. Ball's papers are not yet (December 19th) completed—or rather not yet all published—but he states that he "is prepared to demonstrate that the phenomena presented by these cases are due to an entirely different source from that of rheumatism, and that the symptoms, the progress, and the anatomical characteristics of spinal arthritis, as far as we are acquainted with them, are totally distinct from those attendant on *rheumatic* affections of the joints." We must wait and see—merely venturing the remark that it is very extraordinary that Trousseau and Duchenne, out of all their cases of locomotor ataxia, should have found none with this peculiar complication, while in the course of the last two or three years eleven cases are reported in the hospitals of Paris; extraordinary, we mean, if there is any real connection between these two diseases.—*Richmond Medical Journal.*

---

A KNOT IN THE FUNIS.—At the Pathological Society of New York, (*Med. Record,*) Dr. Noolan presented a portion of a funis 24 inches long, tied in quite a firm knot. The child was born alive.

